# Efficacy of PPV Combined with Air Tamponade for Treatment of Inferior Retinal Breaks

**DOI:** 10.1155/2021/9597584

**Published:** 2021-07-21

**Authors:** Yong Zhang, Xin Li, Guiping Pan, Zhen Tian, Siwei Liu, Jun Yuan

**Affiliations:** Department of Ophthalmology, Taihe Hospital, Hubei University of Medicine, Shiyan, Hubei, China

## Abstract

**Purpose:**

To observe the efficacy and safety of pars plana vitrectomy (PPV) combined with filtered air tamponade in the treatment of rhegmatogenous retinal detachment (RRD) with inferior retinal breaks.

**Methods:**

This retrospective study included 20 patients (20 eyes) with inferior retinal breaks in RRD; all underwent PPV combined with filtered air tamponade. Preoperative examinations included BCVA, IOP, anterior segment, fundus and locations, numbers, and sizes of retinal breaks and ocular B-mode ultrasonography. Postoperative examinations included BCVA, IOP, residual gas volume, retinal reattachment, and complications.

**Results:**

After follow-up for 1 year, the primary retinal reattachment rate was 95% and the final reattachment rate was 100%. Pre- and postoperative BCVA averaged 1.51 ± 0.63 and 0.97 ± 0.58 logMAR, respectively; the difference was statistically significant (*P* < 0.001). Average pre- and postoperative IOP were not statistically different. The average volume of residual gas on the first day after the surgery was 77.5%; the gas was absorbed in all patients within 2 weeks; no significant postoperative complications were observed.

**Conclusion:**

PPV combined with filtered air tamponade is a safe and effective treatment for RRD with inferior retinal breaks. Notably, the retinal reattachment rate is high, gas absorption is rapid, and incidence of complications is low.

## 1. Introduction

Rhegmatogenous retinal detachment (RRD) is characterized by the formation of retinal breaks, which is the separation between the retinal neuroepithelium and pigment epithelium layers [1]. It is a clinically common cause of blindness. Vitreoretinal surgery is the most common surgical method for the treatment of RRD. With the continuous development of vitreoretinal surgery technology, vitreous substitutes have been explored. Commonly used intraocular filling materials include air, inert gas, and silicone oil. An inert gas bubble has strong pressure on the eye, can remain in the vitreous cavity for an extended period, and can be spontaneously absorbed; however, inert gas expands inside the eyes, leading to increased postoperative intraocular pressure (IOP) and potential blindness [2]. Silicone oil cannot be absorbed; thus, it can sustain pressure on retinal breaks but must be removed by an additional surgery. In addition, there are many complications after silicone oil tamponade, such as transiently increased IOP, secondary glaucoma, silicone oil emulsification, anterior chamber inflammation, corneal degeneration, and complicated cataract [3, 4]. In contrast, air does not expand in the eye, exhibits a short retention time in the vitreous cavity, and is readily absorbed and there are fewer postoperative complications. However, air exhibits a short retention time in the vitreous cavity, along with rapid absorption and an associated brief period of pressure on the retina, especially for inferior retinal breaks. Thus, there remains much debate regarding the efficacy and safety of pars plana vitrectomy (PPV) combined with filtered air tamponade in the treatment of RRD with inferior retinal breaks. In this study, we observed the efficacy and complications of PPV combined with filtered air tamponade in the treatment of RRD with inferior retinal breaks, in order to explore the safety and efficacy of PPV combined with filtered air tamponade in the treatment of RRD with inferior retinal breaks.

## 2. Materials and Methods

### 2.1. Clinical Data

Retrospective analysis included data collected from patients RRD with inferior retinal breaks who underwent PPV combined with filtered air tamponade at the Ophthalmology Department of Taihe Hospital in Shiyan City from January 2018 to June 2019. This study was conducted in accordance with the Declaration of Helsinki. All the patients were informed of the nature of their disease and of all potential treatment options. They participated in this study voluntarily without any additional compensation. Informed consent was obtained from all the patients. The study sample comprised 20 patients (20 eyes: 12 right and 8 left), 9 males and 11 females; the patients were aged 21 to 70 years, with an average of (48.10 ± 14.32) years. Their course of disease ranged from 4 days to 30 days, with an average of 13.45 ± 9.00 days. The locations, numbers, and sizes of retinal breaks were determined based on intraoperative findings (Table 1). Macular detachment was determined by preoperative macular optical coherence tomography (OCT) examination. Macular detachment occurred in 20 patients (100%). Exclusion criteria were as follows: proliferative vitreoretinopathy of grade C or greater; a history of large retinal tears; presence of other eye diseases (e.g., glaucoma or macular hole) that seriously affect vision; follow-up period less than 1 year; or patients lost to follow-up. RRD occurred in all 20 cases (20 eyes), among which 5 eyes were pseudophakic and 7 exhibited a high degree of myopia (−6.00 D to −11.00 D).

### 2.2. Methods

Preoperative examination included review of medical history, visual acuity, IOP, anterior segment, fundus, OCT, and B-mode ultrasonography.

All operations were performed by the same surgeon. The standard three incisions on the pars plana were made after the patient underwent application of local anesthesia; a wide-angle viewing system with hand-held infusion lenses was used (field of view: 112° to 134°) to perform PPV, especially the complete removal of the vitreous body surrounding the breaks. After drainage of subretinal fluid (SRF) with gas/liquid exchange, intraocular laser photocoagulation or cryocoagulation under the operating microscope was performed for closure of retinal breaks and areas of degeneration. A flute needle was used to extract SRF, in order to ensure that the vitreous cavity was filled with filtered air. If the SRF could not be easily discharged, heavy perfluoron (PFO) was used intraoperatively to discharge SRF and flatten the retina. After gas/PFO exchange, intraocular laser photocoagulation or cryocoagulation under the operating microscope was performed for closure of the retinal breaks and areas of degeneration. Patients who exhibited lens dislocation were also treated with phakectomy during the operation. A detailed examination was performed to check the closure of all breaks before completion of the operation. For 72 hours after surgery, each patient remained strictly in the prone position, especially during the first 24 hours after surgery, to maintain the inferior retinal breaks at a high position; each patient adopted the prone position for as long as possible per day thereafter until the gas was absorbed.

Postoperative examinations comprised daily examinations for the first 3 days after surgery. Checks of visual acuity, IOP, and the anterior segment and fundus were performed at 1 week, 2 weeks, and at 1, 3, 6 and 12 months after surgery to observe residual gas volume, closure of breaks, and retinal reattachment, as well as new retinal breaks had formed. The residual gas volume was measured as the vertical meniscus height of the bubble in the eye at the slit-lamp with fundus lens (Volk Optical, Inc.) [5].

Efficacy determination mainly comprised anatomical reattachment of the retina at 1 year after surgery. Cure was defined as closed retinal break with the retina completely reattached; improved was defined as completely closed break with SRF remaining; uncured was defined as an unclosed break with detached retina; recurrence was defined as detached retina after it had been reattached.

Best-corrected visual acuity (BCVA) was measured before and after surgery by the same surgeon and was converted to the logarithm of the minimum angle of resolution (logMAR) [6, 7].

Dates were reported as the mean ± SD and analyzed using the paired *t*-test for numerical and ordinal variables. All the tests were two-sided, and *P* < 0.05 was considered statistically significant. Statistical analysis was performed using SPSS 22.0 statistical software.

## 3. Results

Reattachment rate of the retina, visual acuity before and after operation, postoperative IOP, and residual gas volume are shown in Table 2.

For the purpose of this study, an inferior break refers to a break at the lower part of the retina (between 4 and 8 o'clock position). Among all 20 patients, the primary retinal reattachment rate was 95% (19/20) and the final reattachment rate was 100% (20/20). One patient with a pseudophakic eye was treated with PPV combined with filtered air tamponade; visual acuity decreased again at 1.5 months after the first surgery. Mydriasis examination of the fundus showed that the upper, lower, and temporal retina was extensively detached; a new retinal break was observed in the 3 o'clock position, proximal to the ora serrata. Total vitrectomy and fluid-air exchange were performed, and a flute needle was applied to extract the SRF. After the closure of the retinal break by use of a laser, a filtered air tamponade was again performed. The break was closed in this patient and the retina remained flat at 1 year after surgery; in the remaining 19 eyes, no recurrence of retinal detachment was found after surgery.

In this group of patients, preoperative BCVA was 0.2–2.3 logMAR, with an average of 1.51 ± 0.63 logMAR. Postoperative BCVA comprised BCVA at 1 year after operation; it was 0–2.0 logMAR, with an average of 0.97 ± 0.58 logMAR. There was a significant difference in BCVA before and after operation (*P* < 0.001).

Preoperative IOP was 12–23 mmHg, with an average of 17.20 ± 3.05 mmHg; IOP was normal in all patients during postoperative follow-up. IOP was 11–21 mmHg on the first day after surgery, with an average of 16.70 ± 3.08 mmHg. There was no significant difference compared with preoperative IOP (*P*=0.096).

The residual gas volume on the first day after surgery was 60%–90%, with an average of 77.50 ± 9.11%; the residual gas volume on the seventh day after surgery was 0–40%, with an average of 17.50 ± 11.18%. At 2 weeks thereafter, all the gas was absorbed.

### 3.1. Postoperative Complications

Extensive retinal detachment recurred in one patient with a pseudophakic eye at 1.5 months after surgery; a new retinal break appeared in a location that was different from the original break.

During the follow-up period, there were no missing retinal breaks, epiretinal membranes, and residual subretinal fluid.

## 4. Discussion

**Figure 1:**
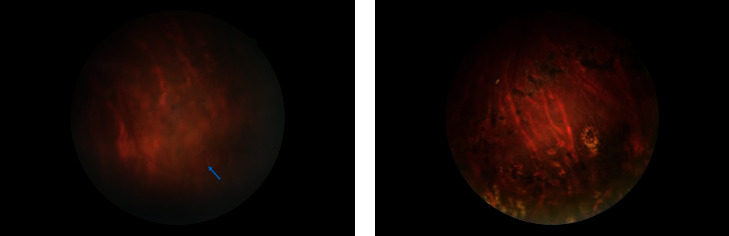
A 25-year-old man presented with retinal detachment in the left eye. (a) The preoperative examination is shown with retinal break. The blue arrow represents the location of the hole. (b) 6 months after surgery, the retina is completely reattached and the retinal break is closed. Laser photocoagulation spots are visible.

The vitreoretinal surgery technique can significantly improve the cure rate of retinal detachment [8]. Vitreoretinal surgery typically requires the use of intraocular filling materials to ensure the flattening of retinal breaks and maintain tight attachment of the detached retina and retinal pigment epithelium (RPE) [9]. Intraocular filling gas maintains retinal attachment through high surface tension and mechanical buoyancy and blocking the production of SRF by preventing intravitreal fluid from passing through the retinal break into the subretinal space [10]. However, the intraocular filling materials serve as a barrier against the entry of intravitreal fluid into the subretinal space; this barrier no longer functions when the SRF is absorbed and an adhesion is formed between the retina and RPE layer [11]. Many studies have shown that the adhesion between the retina and the RPE layer has been formed within 24 hours by blocking the source of SRF [12–15], and our prospective controlled study [16] also proved that complete SRF is unnecessary as residual SRF can be spontaneously absorbed in the 24 hours after surgery. This fully demonstrates the importance of filling material in the first 24 hours after surgery. In addition, a study by Martínez-Castillo et al. [5] showed that there was no statistically significant difference between the remaining volume of air and inert gas on the first day after surgery, indicating that the filtered air as a barrier can achieve the same effect as inert gas in the first 24 hours after surgery. Zhang et al. [17] showed that PPV combined with partial filtered air tamponade (50%) was safe and effective for the treatment of RRD. The average residual gas volume in the vitreous cavity on the first day after surgery was 77.5% in this study. The above results show that the filtered air can provide good pressure and isolation effect to treat retinal reattachment. In this study, breaks were located below the retina in all patients (Figure 1). To ensure the safety of the operation, the patient remained strictly in the prone position for 72 hours after surgery. In particular, in the first 24 hours after surgery, the inferior breaks were maintained in a high position to ensure that the air insulated intravitreal fluid from entering the subretinal space and to promote formation of an adhesion between the retina and RPE layer. Ajlan et al. [10] demonstrated the importance of maintaining the prone position for the first 24 hours after surgery. In addition, the best posture to take in inferior breaks has been confirmed [18].

Tan et al. [19] showed that the retinal reattachment rate of inferior breaks with air tamponade was only 69.6%; inert gas as the filling material demonstrated a higher success rate than air in the treatment of RRD with inferior retinal breaks. However, a recent randomized controlled trial [20] showed that air tamponade can achieve the same effect as C3F8 in the treatment of RRD with inferior retinal breaks; moreover, air is absorbed more quickly, can restore vision sooner, and allows early detection of redetached retinal breaks. In this study, there was a statistically significant difference in BCVA before and after operation (*P* < 0.001). Postoperative visual acuity was improved and patient satisfaction was higher. Martínez-Castillo et al. [21, 22] reported that the primary retinal reattachment rate was no less than 90% and the final reattachment rate was 100% in patients undergoing PPV combined with air tamponade for retinal detachment with inferior retinal breaks in pseudophakic eyes. In our study, the same operation method was used and the prone position was maintained after operation. The retinal reattachment rate after the first operation was 95% (19/20), and the retinal reattachment rate after the final follow-up was 100% (20/20). Our results are largely consistent with those of the prior study. In the current study, one patient developed recurrent retinal detachment at 1.5 months after the first surgery. Examination with three-mirror contact lens revealed the formation of a new break near the base of the vitreous body, whereas the previously treated subnasal retinal break had properly healed. In this position at the base of the vitreous body, residual vitreous body was found. The most common cause of rhegmatogenous retinal detachment is degeneration of the vitreous body [23]. Traction by the residual vitreous body might have led to the formation of the new retinal break. Therefore, complete relief of traction around the retinal breaks is key to a successful operation. The residual vitreous body was resected by a second operation, fluid-air exchange was performed again, SRF was fully drained, and the retinal break and peripheral retinal focal areas were reclosed by the use of a laser. Vitreous bodies surrounding all breaks were completely removed in this study to ensure complete relief of traction around the retinal breaks. Besides, laser photocoagulation or cryocoagulation was applied to the edges of the break, as well as to the area of degeneration, which provided firm adhesion between the retina and RPE layers in patients whose retinas were filled with filtered air. The results of this study showed that PPV combined with filtered air tamponade is safe and effective for the treatment of uncomplicated retinal detachment. Advantages of filtered air tamponade over inert gas tamponade [11] are as follows: inert gas has a higher price and higher requirements for transportation and storage; air is cheap, abundant, and convenient to obtain. Besides, transportation, storage, and dilution of inert gases can result in contamination of the gas, with toxic effects. Inert gas can disturb the vitreous body [24, 25], thus increasing the risk of high IOP and new or missing retinal breaks. Because air is a short-acting gas that is nonexpandable, it causes minimal interference with the vitreous body and requires remaining in the prone position for a shorter period after surgery. Postoperative complications, such as high IOP, are also less common; the results of our study confirm this.

It is safe and effective to treat inferior retinal breaks in retinal detachment with air tamponade. In addition, the use of air as the filling material significantly reduces many complications caused by silicone oil and inert gas and can restore vision and improve the patient's quality of life earlier. The study limitations include the following: mission of large breaks, PVR, and patients lost to follow-up; hence, the 95% success relates to a limited range of inferior RD. However, due to the small sample sizes, the effects must be confirmed by large-scale multicenter clinical studies.

## Figures and Tables

**Table 1:** Locations, numbers, and sizes of inferior retinal breaks.

Break position	Eye	Number of breaks	Size of breaks (PD)
Subtemporal	7	9	1/3–3/4
Subnasal	8	9	1/3–1
6 o'clock position	2	2	1/2–3/4
Superior and inferior	3	8	1/4–1

PD: papillary diameter.

**Table 2:** Postoperative examination data of inferior retinal breaks.

Cure rate after the first surgery	95%
Cure rate after the final surgery	100%
Recurrence	1 eye
*Average BCVA (mean* *±* *SD)*	
Before operation	1.51 ± 0.63 logMAR
3 months after operation	0.97 ± 0.58 logMAR

*Average intraocular pressure on the first day after operation*	
Mean ± SD	16.70 ± 3.08 mmHg

*Residual gas volume*	
First day after operation	77.50 ± 9.11%
Seventh day after operation	17.50 ± 11.18%
Macular status (on/off)	20/0

BCVA: best corrected visual acuity; SD: standard deviation; logMAR: logarithm of the minimum angle of resolution.

## Data Availability

The data used to support the results of this study are available from the corresponding author (inforzy@163.com) upon request.

## Additional Points

*Summary*. This retrospective study evaluated the efficacy and safety of air tamponade in the treatment of RRD with inferior retinal breaks. After follow-up for 1 year, the primary retinal reattachment rate was 95%. Postoperative visual acuity was significantly improved, and prone position time was significantly shortened compared with that before surgery.
